# The assessment of generalized anxiety disorder: psychometric validation of the Spanish version of the self-administered GAD-2 scale in daily medical practice

**DOI:** 10.1186/1477-7525-10-114

**Published:** 2012-09-19

**Authors:** Javier García-Campayo, Enric Zamorano, Miguel A Ruiz, María Pérez-Páramo, Vanessa López-Gómez, Javier Rejas

**Affiliations:** 1 Department of Psychiatry, Hospital Miguel Servet, Zaragoza, 50009, Spain; 2 Primary Care Centre Sant Antoni de Vilamajor, ABS Alt Mogent, Barcelona, Spain; 3 Department of Methodology, School of Psychology, Universidad Autónoma de Madrid, Madrid, Spain; 4 Medical Unit, Pfizer, S.L.U, Alcobendas, Madrid, Spain; 5 Health Economics and Outcomes Research Department, Pfizer, S.L.U, Alcobendas, Madrid, Spain

**Keywords:** GAD-2, Generalised anxiety disorder, Screening, Primary care, Psychometric validity

## Abstract

**Aim:**

To psychometrically validate the Spanish version of the self-administered 2-item GAD-2 scale for screening probable patients with generalised anxiety disorder (GAD).

**Methods:**

The GAD-2 was self-administered by patients diagnosed with GAD according to DSM-IV criteria and by age- and sex-matched controls who were recruited at random in mental health and primary care centres. Criteria validity was explored using ROC curve analysis, and sensitivity, specificity and positive and negative predictive values were determined for different cut-off values. Concurrent validity was also established using the HAM-A, HADS, and WHODAS II scales.

**Results:**

The study sample consisted of 212 subjects (106 patients with GAD) with a mean age of 50.38 years (SD = 16.76). No items of the scale were left blank. Floor and ceiling effects were negligible. No patients with GAD had to be assisted to complete the questionnaire. Reliability (internal consistency) was high; Cronbach’s α = 0.875. A cut-off point of 3 showed adequate sensitivity (91.5%) and specificity (85.8%), with a statistically significant area under the curve (AUC = 0.937, p < 0.001), to distinguish GAD patients from controls. Concurrent validity was also high and significant with HAM-A (0.806, p < 0.001), HADS (anxiety domain, 0.825, p < 0.001) and WHO-DAS II (0.642, p < 0.001) scales.

**Conclusion:**

The Spanish version of the GAD-2 scale has been shown to have appropriate psychometric properties to rapidly detect probable cases of GAD in the Spanish cultural context under routine clinical practice conditions.

## Background

Anxiety disorders are among the most common mental disorders with a prevalence of 12% in the adult population [1]. In the ESEMeD [2] epidemiologic study on mental disorders conducted in six European countries, past-year prevalence was 6% while life time prevalence was 13.6%, with 1.9% prevalence in Spain (2.6% in women and 1.2% in men). Generalised anxiety disorder (GAD) studies in the general population in the United States and Canada have found prevalences in the range of 5-7%. These figures rise in the population visiting primary care health centres, with 19.5% of patients presenting some form of the most common anxiety disorders (GAD, panic disorder, social anxiety disorder or post-traumatic stress) [3]. Approximately 8% of those patients are diagnosed with GAD, making it the most prevalent anxiety disorder [4]. In a World Health Organisation study applying ICD-10 criteria to more than 25,000 primary care patients in 14 countries, GAD prevalence was 7.9%, matching the values found in the Spanish primary care population [5-7].

It is clear that GAD is under-diagnosed and only a minority of cases are detected in primary care services [8], possibly due to the fact that the use of diagnostic questionnaires constitutes a burden on routine practice, because of the impact on consultation time and the associated cost [9]. There is therefore a need for simple questionnaires that can be administered quickly in routine practice and also on Web health portals and for Internet-based studies. The core symptom of GAD is chronic, excessive and uncontrolled worry [10]. Both the DSM-IV and ICD-10 classification systems [11,12] distinguish GAD from other anxiety and depressive disorders [13]. However as is the case with other anxiety disorders, GAD is frequently associated with other psychiatric diseases: 80-90% of individuals meeting GAD criteria present one or more other psychiatric disorders during their lifetime [14]. The effects of GAD on health-related quality of life (HRQL) are even greater than those observed in major depression disorder, which is known to be disabling and costly [15], and it is not possible to totally explain these effects by comorbid diseases.

Several scales for assessing the presence of clinical anxiety have been validated in our culture and are used routinely. These include the Hamilton Anxiety Scale (HAM-A) [16], Hospital Anxiety and Depression Scale (HADS) [17], Covi Anxiety Scale [18], Clinical Anxiety Scale (CAS) [19], State-Trait Anxiety Inventory (STAI) [20], Generalized Anxiety Disorder Questionnaire-IV (GAD-Q-IV) [21], and World Health Organization Composite International Diagnostic Interview Short-Form (CIDI-SF) [22], among others. Regardless of whether they are patient self-administered or structured interviews administered by the clinician, they all assess the presence of anxiety symptoms and their severity, but are not adequate for early detection or screening of probable GAD cases and cannot be self-administered by the patient without clinician supervision. Recently the GAD-7 scale has been developed and validated [23,24]. It is an easy to use 7-item instrument, based on DSM-IV criteria, for identifying probable GAD cases. It has excellent psychometric properties, is easy to administer and does not involve any burden for the patient or for the clinician. Furthermore its shortness allows it to be used in epidemiologic studies and also in remote health surveys along with other health questionnaires. However it has been suggested that the questionnaire be further shortened to use only the first two items related to the two main GAD symptoms, whence the GAD-2 scale [3]. The GAD-2 questionnaire has been proven to retain the good psychometric properties of the extended version and to have similar discriminant capability [3,25]. Given the importance of early detection of anxiety disorders, the GAD-2 has been proposed as a common first step for screening GAD [3].

The aim of the present research was to develop a new version of the GAD-2 questionnaire, culturally adapted into Spanish for Spain and to assess its psychometric properties for screening probable subjects with GAD in daily medical practice.

## Material and methods

The development of the GAD-2 cultural adaptation was approached as an extension of the broader adaptation of the GAD-7 scale, since items are shared with the latter. The methodology used was the one currently recommended for adapting psychometric instruments [26,27] along with assumptions of classical test theory [28]. The GAD-7 adaptation process started with a double translation from English followed by back-translation of the adapted instrument. The process of the questionnaire cultural adaptation was initiated with the duplicate translation of the English original into Spanish by two English-speaking native translators separately. Both translations were reviewed by an Expert Panel including 4 clinicians (one of them a psychiatrist), 1 expert in clinical research and 2 methodologists specialized in measurement tasks. Then, both translations were merged into a single reconciled version resolving discrepancies by full agreement, which underwent a content validity process by inter-rater agreement estimation. For that purpose, a panel of 8 specialists in psychiatric disorders was selected. These specialists independently assessed whether each item did or did not properly measure GAD (objective concept) and whether it could or could not measure depression (distractor concept). The item-objective congruence index was computed from the specialists’ ratings [29]. This index reaches the value of 1 in case of perfect congruence in assigning the item to one domain, and −1 when such congruence is lacking. The conciliated version was administered to the pilot sample together with a brief additional questionnaire to ascertain the help needed to complete the questionnaire, the difficulties encountered and socio-demographic variables. In view of the results obtained in the pilot test, the questionnaire header was modified to emphasize the frequency of symptom onset; the anchors of the response categories were also modified. The final version was translated back into English by two independent translators separately and forwarded to the original authors for conceptual equivalence assessment. The structural validity was assessed through confirmatory factor analysis, concurrent validity through the relation with the HAM-A and HADS scales, and construct validity through the relation with the WHODAS II scale [30]. The entire process has been reported elsewhere [24].

Subsequently a second measurement study was performed, administering the GAD-7 in conjunction with the GAD-2 in order to determine whether there might be differences between the extended and the abbreviated versions. If differences do exist, we would like to be able to recommend that researchers use the scale version more suited to their research needs, based on accuracy and length. Furthermore it would not be advisable to use the information gathered in previous studies using the GAD-7 to deduce outcomes that would have been obtained had the GAD-2 been used as a screening tool.

### Study design

The present study was designed as a multicentre, observational, cross-sectional study conducted under routine clinical practice conditions in primary care clinics, in subjects diagnosed with GAD according to DSM-IV criteria. Participating clinicians were family physicians trained in applying DSM criteria and in the use of instrument measuring mental health (for example, Hamilton scales) with more of 5 years of experience in visiting outpatients at primary care settings. Standard diagnosis of GAD was based in the Mini International Neuropsychiatric Interview. All subjects in the study – both patients and controls – had to give their informed consent in order to be enrolled in the study and have their data analysed. The study protocol was approved by the Research Ethics Committee of the Universidad Autónoma de Madrid and by the Research Committee of the Spanish Society of Rural and General Medicine (SEMERGEN). At the time of recruitment, the GAD-7 questionnaire was administered to the sample of patients and controls along with two other instruments: the Spanish versions of the HAM-A [19] and the WHODAS II (WHO 2000) [30]. Socio-demographic and medical record data were also recorded at this visit.

In a second stage the concordance between the GAD-2 and GAD-7 was gauged in order to determine whether the GAD-2 could have been used as a diagnostic tool in studies were the GAD-7 had been used. The GAD-2 was administered to a new sample composed of 60 patients. This stage was also needed to assess the psychometric capabilities of the GAD-2 alone. The sample was randomly divided in two parts. In one part the GAD-2 was presented before the GAD-7 and in the second part the presentation order was reversed. Using this design it would be possible to control for a trailing effect due to presentation order.

### Sample of patients

Patients were randomly screened in primary care settings in urban areas of Madrid, Zaragoza and Barcelona during office visits with study investigators. Once screened, patient fulfilment of the following inclusion criteria was checked: patients of either sex; over 18 years of age; able to speak and understand Spanish; with a known diagnosis of GAD (for the patient group, a diagnosis according to DSM-IV criteria under usual medical practice conditions was required), either receiving no anxiolytic treatment of any type or receiving anxiolytic therapy but with anxiety symptoms still present (score ≥ 16 points on the HAM-A). Likewise the following exclusion criteria were verified: patients or subjects who, in the investigator’s judgment, were in a state of health that would not allow self-administered completion of self-perceived health scales; were unable to understand or answer the questions on the scale due to their educational level or lack of knowledge of the Spanish language; or were under pharmacological treatment likely to interfere with their ability to understand and answer the questions on the scale. Once a case was identified and enrolled in the study, a control subject was selected concurrently at the participating centre from among those subjects attending the clinic for any reason unrelated to an anxiety disorder. Thus controls were age- (± 5 years) and sex-matched subjects without a diagnosis of anxiety disorder of any kind and no symptoms of anxiety (HAM-A < 10). Sample size was estimated with respect to sensitivity of the GAD-7 scale for screening possible cases of the target disease (GAD). We needed 100 participants with GAD to ensure that the total width of the 95% confidence interval around a sensitivity proportion of 0.90 was no greater than 0.05, given that the estimated prevalence of GAD in clinic populations in Spain ranges between 6% and 8% (ESEMeD/MHEDEA) [2,7]. A similar sized age- (± 5 years) and sex-matched control group (without GAD) was also enrolled. We increased the sample size by 5% to allow for losses of information in the statistical analysis. Patients were randomly selected as they attended consultation by 14 investigators (family doctors in urban zones in the provinces of Madrid, Zaragoza and Barcelona). In the end, the sample comprised a total of 212 individuals, half in the GAD group and the other half in the control group.

The GAD-2 vs GAD-7 concordance sample was recruited following the same inclusion/exclusion criteria used in the cultural adaptation sample. A sample of 60 patients was assumed to be sufficient to estimate the correlation between scales.

### Concurrent instruments

The 7-item generalised anxiety disorder scale (GAD-7) is a self-administered instrument whose overall score is computed by addition of the item scores (see Table 1). Each of the 7 component items can be assigned one of the following values: 0 (never), 1 (several days), 2 (more than half of the days) or 3 (almost every day). Theoretical overall score can range between 1 and 21 points and may be used to assign patients to one of the following severity levels: minimal (0 – 4), mild (5 – 9), moderate (10 – 14) or severe (15–21). The questionnaire has excellent reliability and discriminant properties: Cronbach’s α = 0.92; area under the curve (AUC) = 0.91. Using a cut-off score of ≥10, a sensitivity = 0.86 and specificity = 0.82 have been obtained in primary care patients [3]. General population norms have been published [31].

**Table 1:** GAD-7 and GAD-2 items

**Spanish**	**English**	**Scale**
Sensación de nerviosismo, de ansiedad, de tener los nervios de punta	Feeling nervous, anxious, or on edge	GAD-7, GAD-2
Incapacidad para eludir o controlar la preocupación	Not being able to stop or control worrying	GAD-7, GAD-2
Preocupación excesiva por cosas diferentes	Worrying too much about different things	GAD-7
Dificultad para relajarse	Trouble relaxing	GAD-7
Una intranquilidad de tal grado que no puede estarse quieto	Being so restless that it is hard to sit still	GAD-7
Facilidad para enfadarse o irritabilidad	Becoming easily annoyed or irritable	GAD-7
Sensación de miedo, como si pudiera ocurrir algo terrible	Feeling afraid as if something awful might happen	GAD-7

The 2-item generalised anxiety disorder scale (GAD-2) is composed of the first two items of the GAD-7 and they gather information on the two core GAD symptoms (see Table 1). The overall score is obtained by simple addition of item scores. Each individual item can be scored between 0 (never) and 3 (almost every day). The overall score can range between 0 and 6 and can be used to assign patients to the following severity levels: minimal (0–2) and severe (3–6). Using a cut-off value ≥3, a sensitivity = 0.86, specificity = 0.83, positive predictive value (PPV) = 0.34 and negative predictive value (NPV) = 0.94 were obtained when Web-based administration methods were used, along with an AUC = 0.91 [3]. Using a cut-off value ≥4, the Web-administration discrimination values obtained were: sensitivity = 0.83, specificity = 0.61, PPV = 0.34, NPV = 0.94 and AUC = 0.76 [25].

The Hamilton Anxiety Scale (HAM) [16] is a 14-item, hetero-administered scale formulated as a semi-structured interview to assess the subject’s level of anxiety. Items are scored from 0 (absent) to 3 (severe). The total score ranges from 0 to 42 points and may be categorized into four severity groups: normal (0 – 9), mild (10 – 15), moderate (16 – 24) and severe (25 – 42).

The Hospital Anxiety and Depression Scale (HADS) [17] is a 14-item, self-administered scale with Anxiety and Depression being assessed by 7 items each. Each item is scored from 0 to 3 with several anchors. Some items are assessed positively and others negatively. A score between 0 and 21 points may be obtained in each domain. The score in each domain may be categorized into four severity groups: normal (0 – 7), mild (8 – 10), moderate (15 – 21) and severe (15 – 21).

The World Health’s Organization Disability Assessment Scale (WHO-DAS II 12 items version) [30] is a 12-item, self-administered scale. Items are grouped by pairs in 6 domains: 1- Understanding and communicating with the world, 2- Moving and getting around, 3-Self care, 4-Getting along with people, 5-Daily life activities (domestic responsibilities, leisure, and work), and 6-Participation in society. This scale contains another 5 items, one about overall health and four about the number of days with activity limitations in daily life. Scoring is standardized on a 0–100 metric, where 0 means no disability and 100 the highest disability.

### Statistical analysis

Firstly item analysis was performed by computing the selection frequency for each response category, along with non-responses. Reliability was assessed by computing internal consistency using Cronbach’s alpha [32] and the correlation between the GAD-2 items and those same items extracted as a subscale from the GAD-7. The intra-class correlation coefficient (ICC) for individual measures was computed to prevent questionnaire length from affecting the estimates. GAD-2 scoring discriminative capability was assessed by dividing the study sample into four quartile groups based on the GAD-7 total score and comparing the first and fourth quartile group mean scores on the overall score and also on the individual item scores. These comparisons could be considered an estimate of GAD-2 discriminant validity.

An adapted version of the multiple indicator multiple cause (MIMIC) model was estimated using structural equation modelling [33] in order to determine whether there might be an effect of presentation order (GAD-2 first vs. GAD-7 first) on the mean GAD-2 value when comparing the estimated mean true score for the GAD-2 (T-GAD2) and the mean true score obtained using the extracted GAD-7 subscale (T-GAD7). The model estimates the effect (γ_ij_) of presentation order (0 = GAD-2 first, 1 = GAD-7 first) in terms of deviation from the mean reference value. If no significant effects were found we would be able to conclude that there is no order effect. The model also estimates the measurement capabilities of the individual items (λ loadings) for gauging the true score, whether presented in the GAD-2 alone or the extracted GAD-7 subscale. Measurement loadings should be similar. Raw estimates are reported in order to represent item mean values and error variance estimates and because of the known bias introduced by the regular standardisation method [34]. The discriminant capabilities of the GAD-2 were assessed using the receiver operating characteristic (ROC) curve statistics, against the clinician diagnosis using DSM-IV criteria. AUC was estimated, along with sensitivity, specificity, PPV and NPV for each possible cut-off point value. The ROC curve was compared with those for the GAD-7, HADS, HAM-A and WHODAS II scales. AUC for the GAD-2 was statistically compared against GAD-7 using the method proposed by Hanley and McNeil [35]. Correlations of GAD-2 with other measures were computed, i.e. GAD-7, HADS-Anxiety, HADS-Depression, HAM-A, WHODAS II overall score, WHO-DAS II Dimensions and number of visits to the clinician in the last month.

All computations were done using IBM SPSS Statistics for Windows v19.0 and AMOS 18.0 software [36,37].

## Results

Table 2 summarises the main characteristics of the cultural adaptation and the concurrent measurement samples. The latter comprised 62% women with an average age of 37 years (SD = 10.7, min = 20, max = 58). GAD time since diagnosis varied between 11 months and 23 years with an average of 6.9 years (SD = 5.6).

**Table 2:** Summary and comparison of socio-demographic and clinical variables by study group

	**GAD Group (n = 110)**	**Control Group (n = 110)**	**p**	**Reliability (n = 60)**
Age (years): mean (SD)	47.1 (15.6)	48.0 (16.1)	0.679	37.2 (10.7)
Gender (% women)	72.6	72.6	1.000	62.1
BMI (kg/m^2^)	26.1 (4.8)	25.8 (4.2)	0.635	24.9 (3.4)
Race (% white)	98.1	100	0.498	100
Education (%)			0.262	
None	5.7	1.9		0
Primary	35.8	30.2		21.7
Secondary	24.5	29.2		38.3
Professional Degree	17.0	13.2		13.3
Higher	17.0	25.5		26.7
Marital Status (%)			0.596	
Single	18.9	15.2		28.3
Married	67.0	65.7		43.3
Separated/Divorced	6.6	8.6		26.7
Widowed	7.5	8.6		1.7
Other	0	1.9		0
Labour Status (%)			0.095	
Home-maker	17.9	22.9		10.0
Active	58.5	62.9		45.0
Unemployed	2.8	4.8		33.3
Disabled	10.4	1.9		8.3
Retired	10.4	7.6		3.3
People in Treatment (%)	79	20	< 0.001	NR
Number of Treatments	1.30 (0.11)	0.06 (0.03)	< 0.001	NR
Disorder history (years)	3.49 (0.40)	NA	NA	6.87 (5.56)
HAM	26.54 (9.11)	6.74 (5.12)	< 0.001	NR
HADS-A	12.59 (4.41)	4.08 (2.40)	< 0.001	NR
HADS-D	8.84 (4.99)	2.51 (2.85)	< 0.001	NR
GAD-7	13.96 (4.19)	3.54 (3.32)	< 0.001	13.15 (4.46)
GAD-2	4.58 (1.31)	1.21 (1.39)	< 0.001	3.77 (1.47)
WHODAS-II (overall)	35.78 (22.3)	8.94 (11.8)	< 0.001	NR

*NA* = Not applicable, *NR* = Not recorded, *HAM-A* = Hamilton Anxiety scale, *HADS* = Hospital Anxiety Depression Scale, *GAD-7* = 7-item Generalised Anxiety Disorder scale, *GAD-2* = 2-item Generalised Anxiety Disorders scale, *WHODAS II* = World Health Organisation Disability Assessment Scale II. Data expressed as mean (standard deviation) or percentage.

### Feasibility

None of the GAD-2 items were left blank. Hence, no item comprehension problems were found and issues on pertinence were also not found during the validation process. In the concurrent measurement sample only one individual left one of the GAD-7 items blank, i.e. item 4 (Trouble relaxing). In general responses were spread over all the response categories and the existence of a floor or ceiling effect was discarded. In all items the most frequent response category was the middle one. The percentage of responses in the higher categories varied between 18% and 32% and the percentage of responses in the lower categories varied between 0 and 3%. The distribution of responses on the GAD-2 was similar with 20-25% of responses in the highest category and 2-5% in the lowest category. All items were distributed with a roughly symmetric shape and a skewness index between 0.393 and 0.295 (SE = 0.311).

### Reliability

In the version concordance sample, Cronbach’s alpha for the GAD-2 was 0.927, which is close to the value of 0.936 obtained in the cultural adaptation sample. The ICC for individual measures was 0.707 with an associated 95% confidence interval of (0.554-0.814), while for the GAD-7 it was 0.644 and (0.547-0.739). Regarding the stability of instrument, the mean time between administrations of the GAD-2 and the GAD-7 was 5.9 days (SD = 2.11). When the GAD-2 was administered first, the correlation between GAD-2 items and the extracted GAD-7 subscale was high and significant (r = 0.898, p < 0.001). The estimated reliability for the GAD-2 was α = 0.879 and for the extracted GAD-7 subscale was α = 0.826. The mean value for the GAD-2 (mean = 3.80, SD = 1.58) was slightly lower than for the extracted GAD-7 subscale (mean = 3.97, SD = 1.47), but differences were not significant (dif = −0.17, p = 0.202). When the GAD-2 was presented second, the correlation between formats was again high and significant (r = 0.837, p < 0.001), while the reliability for the GAD-2 was slightly higher (α = 0.780) than for the extracted GAD-7 subscale (α = 0.695). Again the slight difference between GAD-2 mean = 3.73 (SD = 1.36) and extracted GAD-7 subscale mean = 3.97 (SD = 1.35) was not significant (dif = −0.23, p = 0.109).

Comparing the presentation format subsamples, no significant differences were found in the GAD-2 mean score (dif = 0, SE = 0.365, p = 1.00) nor in the extracted GAD-7 subscale (dif = −0.07, SE = 0.382, p = 0.86) nor in the GAD-7 overall score (dif = 0.23, SE = 1.16, p = 0.85). In the cultural adaptation sample, the test-retest correlation between GAD-2 scores (extracted from the GAD-7) was 0.744.

### Presentation order effect

**Figure 1:**
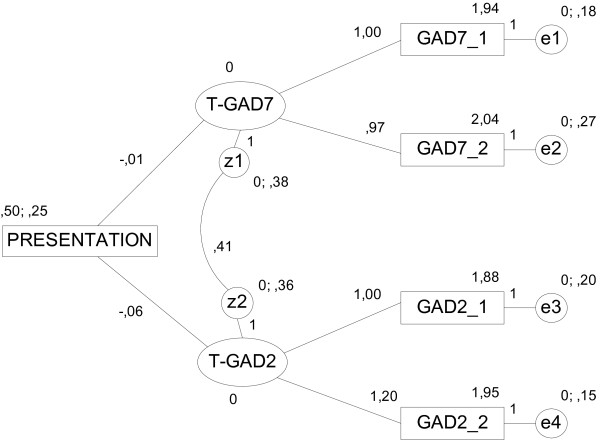
** Structural equation model estimating the impact of presentation order format (GAD-2 first = 1, GAD-7 first = 0) on true GAD mean scores.** T-GAD7 = True GAD-2 subscale score extracted from the GAD-7 scale, T-GAD2 = True GAD-2 score. GAD7_1 & GAD7_2: observed GAD-7 items. GAD2_1 & GAD2_2: observed GAD-2 items.

Figure 1 represents raw estimates of the structural equation model estimating the effect of the presentation format (GAD-2 first vs GAD-7 first). The model attained a good fit with a non-significant value for χ^2^ = 5.48 (df = 3, p = 0.140) indicating that the model is able to properly reproduce the observed means, variances and covariances for the 4 items. Most goodness-of- fit-statistics were adequate: χ^2^/df = 1.83, CFI = 0.984, NFI = 0.966, while the RMSEA = 0.118 was slightly poor. All estimated weights were significant (p < 0.001). Weights associated with the presentation order effect (γ_11_ = −0.01 and γ_21_ = −0.06) on the true mean scores (T-GAD7 and T-GAD2) were not significant (p = 0.936 and p = 0.742, respectively), which allows us to conclude that there is no presentation order effect.

### Discriminant validity

**Figure 2:**
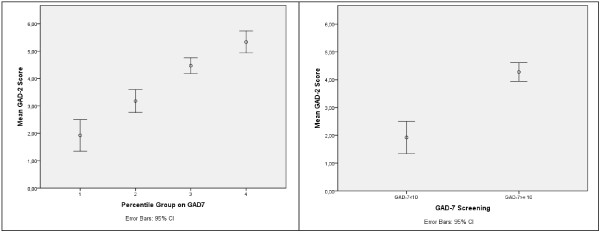
** GAD-2 mean and 95% confidence interval (CI) by percentile group (left) and by GAD-7 screening (right).** Concurrent measurement sample.

Comparing quartile 1 and 4 groups created based on GAD-7 scores, both GAD-2 items showed significant differences (p < 0.001) as did the overall score (see Figure 2). Variances could be assumed to be similar in both groups, although item 1 was clearly homoscedastic (p = 0.843) while item 2 was close to rejection of the equal variances hypothesis (p = 0.075). We can conclude that both items are able to discriminate between low and high scores and both items contribute in the overall score to discrimination. In the concurrent measurement sample, the GAD-2 overall score was able to detect significant differences between GAD screened individuals (GAD-7 ≥ 10: mean = 4.28, SD = 1.14) and non-GAD individuals (mean = 1.92, SD = 0.95). Differences between mean scores were significant (p < 0.001) while variances were similar (p = 0.122; see Figure 2). In the cultural adaptation sample, the value for the area under the ROC curve for the GAD-2 was AUC = 0.918 (SE = 0.037) allowing us to reject the null hypothesis of 0.5 (which would have indicated that it was not possible to discriminate between GAD and control individuals). The 95% confidence interval was (0.846-0.991) indicating excellent discrimination between groups. Using a cut-off value of 3, a sensitivity of 84.6%, a specificity of 87.5%, PPV = 97.8% and NPV = 46.7% were achieved (see Table 3).

**Table 3:** GAD-2 discriminant values for different cut-off points

**Cut-off**	**Sensitivity**	**Specificity**	**PPV**	**NPV**	**Cases**
0	100	0	50	50	212
1	99.1	39.6	62.1	97.7	169
2	97.2	67.0	74.6	95.9	138
3	91.5	85.8	86.6	91.0	112
4	84.9	92.5	91.8	86.0	98
5	56.6	96.2	93.8	68.9	64
6	29.2	98.1	93.9	58.1	33

Note: *PPV* = Positive Predictive Value, *NPV* = Negative Predictive Value. Values are%.

### Screening

**Figure 3:**
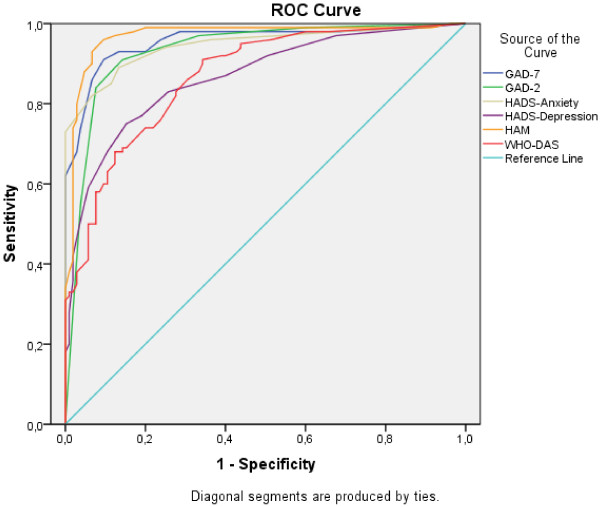
ROC curve for the GAD-2 and concurrent scales.

Figure 3 represents ROC curves for the behaviour of different diagnostic scales with respect to clinician diagnosis. AUC for the GAD-2 was 0.937 (95% CI: 0.902 – 0.971) which includes the central value obtained for the GAD-7 (0.957, SE = 0.015), HAM-A (0.969, SE = 0.013) and HADS-A (0.946, SE = 0.016) but not for the WHODAS II (0.868, SE = 0.024) nor HADS-D (0.867, SE = 0.025). GAD-2 AUC was not significantly different from the one obtained using the GAD-7 (χ^2^ = 3.34, df = 1, p = 0.0675). The best discrimination cut-off value for the GAD-2 was ≥3 with the following discrimination indexes: sensitivity = 91.5%, specificity = 85.8%, PPV = 86.6% and NPV = 91.0%. GAD-2 correlation with other scales was high and significant in all cases (p < 0.001): GAD-7 (0.940), HAM-A (0.806) and HADS-A (0.825), except with WHODAS II (0.642) which was comparatively lower (see Tables 4 and 5).

**Table 4:** GAD-2 correlations with other concurrent scales

	**GAD-2**	**GAD-7**	**HADS-Anxiety**	**HADS-Depression**
GAD-7	0.940^**^			
HADS-Anxiety	0.825^**^	0.903^**^		
HADS-Depression	0.650^**^	0.706^**^	0.724^**^	
HAM	0.806^**^	0.852^**^	0.833^**^	0.756^**^

**Correlation is significant at the 0.01 level (2-tailed).

**Table 5:** GAD-2 correlations with disability measures

	**GAD-2**	**WHODAS**	**Communication**	**Movement**	**Care**	**Interpersonal**	**Work**	**Social**
WHODAS	0.642^**^							
Communication	0.612^**^	0.911^**^						
Movement	0.406^**^	0.809^**^	0.692^**^					
Care	0.361^**^	0.796^**^	0.708^**^	0.690^**^				
Interpersonal	0.533^**^	0.795^**^	0.694^**^	0.471^**^	0.571^**^			
Work	0.587^**^	0.919^**^	0.800^**^	0.722^**^	0.648^**^	0.636^**^		
Social	0.702^**^	0.905^**^	0.806^**^	0.659^**^	0.632^**^	0.680^**^	0.812^**^	
Number of visits	0.438^**^	0.585^**^	0.519^**^	0.539^**^	0.331^**^	0.374^**^	0.531^**^	0.438^**^

**Correlation is significant at the 0.01 level (2-tailed).

## Discussion

The results obtained support the hypothesis that the GAD-2 is a reliable and valid measure for the screening of GAD, both when it is administered alone and when it is extracted as a subscale from previous GAD-7 administrations. Despite the brevity of the scale, good internal consistency ensures that GAD core symptoms are well assessed by the GAD-2. High test-retest correlation reveals good stability over time, suggesting that we are measuring a steady trait in patients as long as no treatment is initiated. The scale is well understood, easy to answer and faster to complete than other questionnaires, demonstrating its high feasibility. GAD-2 scores are able to discriminate between individuals with high and low GAD symptom scores as shown by quartile group comparison. Construct validity is evidenced by the ability to differentiate between patients and controls when detection is done by the clinician and also when other diagnostic scales are used. Predictive validity is confirmed by the good value obtained for the area under the curve, which is even better than values obtained in other samples [25]. Furthermore the GAD-2 screening feature is similar to the extended GAD-7. Convergent validity was proven by the high correlation with other diagnostic scales: GAD-7, HAM-A and HADS, while divergent validity was revealed by the relatively lower correlation with the WHODAS II.

The estimated structural equation model confirmed that the GAD-2 true mean score remained stable regardless of presentation order (before or after the GAD-7), and the GAD-2 score was not favoured by prior administration of the GAD-7. Furthermore GAD-2 values may be extracted from previous studies where the GAD-7 was used without loss of accuracy. Goodness-of-fit statistics were excellent even though this was not the central issue in the model, which only intended to obtain estimates of the presentation order effect. Although it is not reported here, a similar confirmatory model was estimated to assess the stability of the GAD-2 true score regardless of whether it was measured alone or in conjunction with the rest of the GAD-7 items, supporting the hypothesis that the items are stable in both situations (*tau* equivalent measures). Some authors have proposed further reducing the GAD-7 and leaving it condensed into one single item [25], as it has been shown with other instruments, just to facilitate its use at primary care levels [38]. However, this is a controversial matter. In our opinion, that is consistent with previous research also considering that one item may not be as good as longer screeners [38-41], this strategy is not appropriate. Firstly because, from a psychometric perspective, measurement by a single item does not distinguish the variance attributed to measurement error from true score variability. Secondly the proposal by these authors is based on selecting the item with the highest predictive properties by testing regression models containing all GAD-7 items. This analytical strategy does not ensure the psychometric properties of the individual item selected nor does it take into account the context effect of answering the other items present in the scale, a topic we have been able to explore. And finally, because the differential burden of administration of a scale with two or one item seems negligible.

Given the good screening properties of the GAD-2, we recommend its use as a screening tool for early detection of this disorder. However it seems reasonable to prefer the GAD-7 to assess the effect of pharmacological or psychotherapeutic treatments and the follow-up of patients, since it has broader psychometric properties and more sensitivity. It should be taken into account that typical screening processes will question the presence of more than one psychological and physical problem, and could convey administering more than one specific questionnaire (eg.: Minimental for dementia, HADS for depression, etc.) and a whole battery of tests could be desirable. In such situations brief screening instruments should show to be valuable tools. It is important to remember that this instrument is available to all professionals and it can also be accessed on-line on Web health portals, which may be helpful given the high prevalence and comorbidity of this disorder. Nevertheless it should be noted that the screening cut-off points for Web-based administration (both for the GAD-2 and the GAD-7) have been slightly higher than the paper and pencil version [25], although we have not assessed Web-based properties in our own study.

One shortcoming of the present study is that measurements have been carried out in a primary care environment and other authors have shown that GAD-7 scores are slightly higher in this context compared with a general population context (means of 5.57 and 2.95, respectively) [31]. Another limitation of our study is that we did not study the comorbidity influence of other psychological disorders that might be present such as other anxiety disorders or major depressive disorders. We have seen that that a moderate correlation between GAD and Depression scores exists (see Table 4) accounting for 42% of common variance. This limitation has been found before and some authors suggest incorporating an additional question assessing depressed mood, which could be added when a person scores positive on the GAD-7 to distinguish between GAD and Depressive Disorder [25]. Even more when depression diagnostic tools like HAM-D have shown to be able to have detection capabilities for GAD (AUC = 0.867) although they behave significantly worse than GAD specific instruments (p = 0.0062). Given the high level of co morbidity within mental health disorders, a stepwise screening procedure is recommended in order to screen for psychiatric disorders when using self-reported instruments. A short questionnaire screening for the suspected disorder could be used as a first step. If subjects score positive on the specific subscale, they can then undergo more in-depth screening with the appropriate longer questionnaire or diagnostic interview. This type of screening is being investigated [42].

## Conclusion

In summary, despite the identified limitations, the Spanish for Spain version of the GAD-2 scale has been shown to have appropriate psychometric properties for rapidly detecting probable cases of GAD in the Spanish cultural context under conditions of routine clinical practice.

## Competing interests

Vanessa López-Gómez, María Pérez and Javier Rejas are full-time employees of Pfizer, S.L.U. Miguel Ruiz is a professor at the Universidad Autónoma de Madrid which was engaged to perform analysis of data and drafting of a preliminary version of the manuscript. All other authors declare that they do not have conflicts of interest as a consequence of this paper.

## Authors’ contributions

The authors of this manuscript state that they all contributed substantially to manuscript preparation, interpretation of results or study design and logistics. JGC, EZ, MR, VLG, MP and JR were responsible for the study design. JGC and EZ participated in data collection, interpretation of data and manuscript drafting. MR performed data analysis and interpretation. MP and VLG participated in interpretation of data and drafting of the manuscript. All authors approved the final manuscript.

## Funding

Data collection and logistics of the study were funded by Pfizer, S.L.U. Analysis of data and drafting of a preliminary version of the manuscript were funded by Pfizer Inc.
